# An Unusual Presentation of Crohn’s Disease in an Elderly Patient: A Report of a Rare Case

**DOI:** 10.7759/cureus.57977

**Published:** 2024-04-10

**Authors:** Ozoemena Akah, Rakahn Haddadin, Pinak Shah

**Affiliations:** 1 Internal Medicine, MountainView Hospital, Las Vegas, USA; 2 Medicine, MountainView Hospital, Las Vegas, USA

**Keywords:** chronic abdominal pain, internal medicine specialist, elderly onset crohn’s disease, adult gastroenterology, crohn’s disease (cd)

## Abstract

Crohn's disease is a chronic inflammatory bowel disease that primarily affects the terminal ileum and proximal colon. The exact cause is unknown but likely involves genetic factors, environmental triggers, and immune dysregulation. This case report delineates the choice of diagnostics for a 70-year-old patient presenting with symptoms indicative of small bowel obstruction. Initial assessments and imaging suggested a common clinical scenario, yet further investigation uncovered an unexpected diagnosis of Crohn's disease, a condition infrequently encountered in this age demographic.

## Introduction

Crohn's disease (CD), a chronic inflammatory disorder, manifests as a heterogeneous spectrum of gastrointestinal (GI) symptoms, characterized by transmural inflammation of the bowel wall [1]. Patients often experience abdominal pain, diarrhea, weight loss, and fatigue [2,3]. The condition typically involves the terminal ileum and colon but can affect any part of the GI tract [4]. Despite its recognized predilection for younger adults, the incidence of CD in the elderly is rising, albeit less frequently observed in this age group.

Typically diagnosed in individuals between the ages of 20 and 40, the emergence of Crohn's in older populations, especially those over 60 or 70, remains a clinical rarity [5,6]. This demographic shift challenges the conventional diagnostic paradigms as older patients may present with atypical or subtle symptoms, potentially complicating accurate and timely diagnoses.

An initial presentation of small bowel obstruction (SBO) or terminal ileitis is a rare phenomenon, especially in elderly patients. Terminal ileitis is defined as an inflammatory condition of the distal portion of the ileum. There are multiple etiologies that can cause terminal ileitis, with CD being the most common perpetrator of the condition [7]. The clinical presentation often masquerades as mechanical obstruction, leading to initial presumptions favoring surgical causes and delaying consideration of less common etiologies such as Crohn's.

The elderly demographic poses unique challenges in diagnosing GI disorders due to various comorbidities and altered disease presentations. Moreover, elderly patients with CD may exhibit distinct features and atypical symptoms compared to their younger counterparts, necessitating a nuanced approach to diagnosis and management.

## Case presentation

This case involves a 70-year-old male with a complex medical history, including type 1 diabetes mellitus (T1DM), coronary artery disease (CAD) with two prior percutaneous coronary interventions (PCI), and a history of cerebrovascular accident (CVA). The patient initially presented with nonspecific GI symptoms including abdominal pain, which was initially diagnosed as mild SBO or distention with distal ileitis. He was able to pass gas and tolerated diet advancement, leading to his discharge.

However, two weeks later, the patient returned to the emergency department with severe abdominal pain and coffee-ground emesis, accompanied by a sudden onset of sharp and constant pain rated at 10/10. He did not have a bowel movement for two days and had pressure ulcers on his upper back, sacral area, and left elbow, which was worsening.

**Figure 1:**
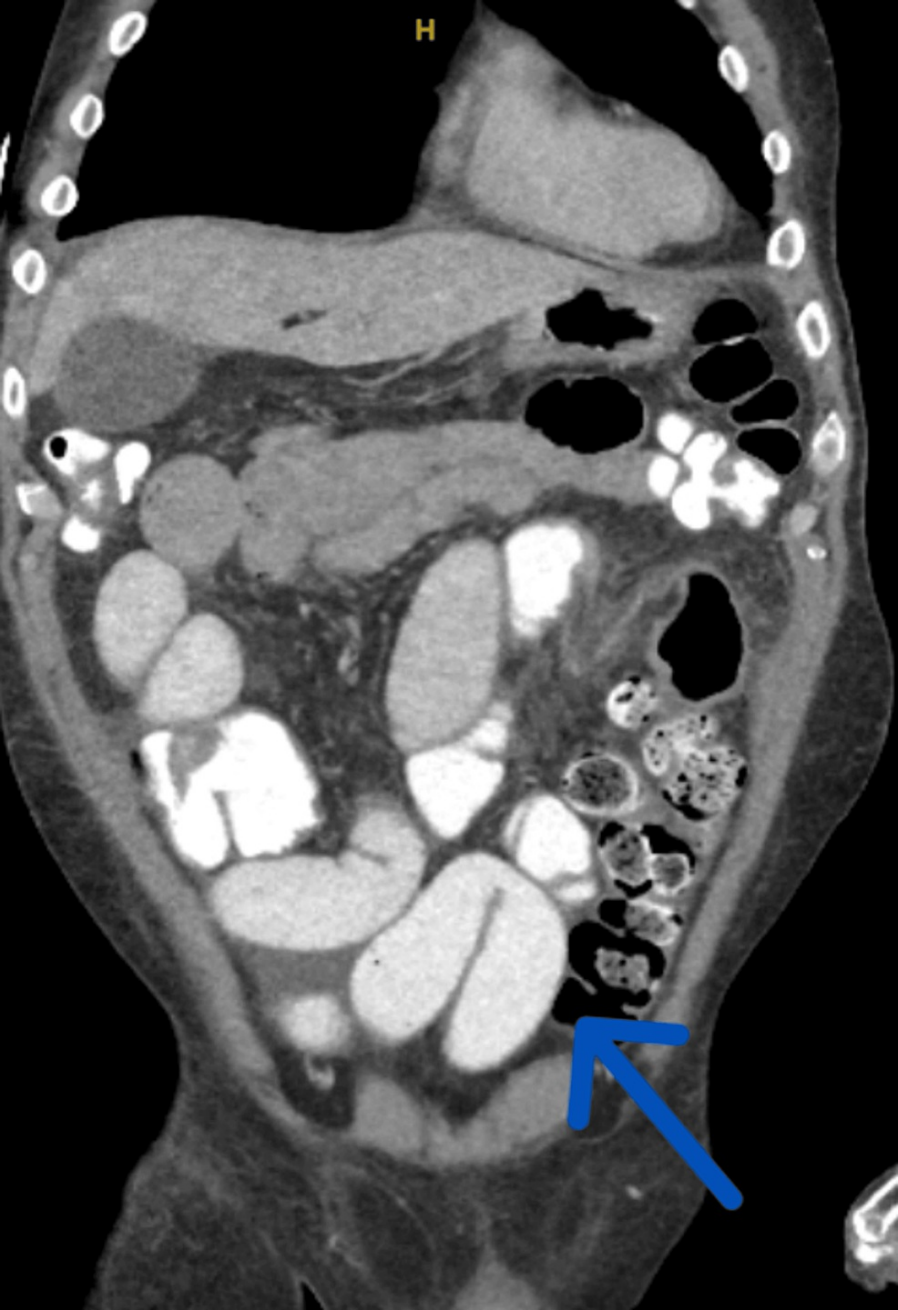
CT scan of the abdomen showing possible ileitis

Physical examination revealed signs of diffuse tenderness and distention. Laboratory findings showed a C-reactive protein (CRP) of 13.10 mg/dL, and an erythrocyte sedimentation rate (ESR) of 88 mm/hr. Imaging studies indicated an SBO with an uncertain transition point and thickening of the terminal ileum, suggestive of terminal ileitis (Figure 1). Conservative measures, including pain management and nasogastric (NG) tube insertion for decompression, were initiated, but the patient's symptoms worsened.

**Figure 2:**
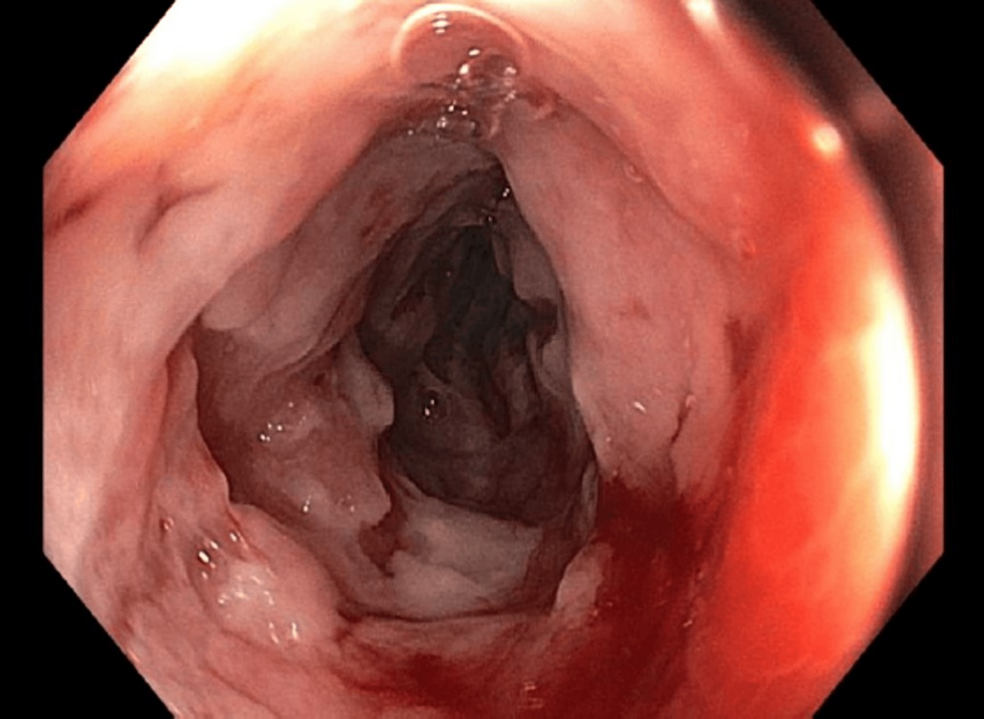
Terminal ileum showing ileitis

A colonoscopy confirmed thickening of the terminal ileum with ulcers, and a biopsy revealed severe active ileitis without any granulomas (Figure 2). Anti-Saccharomyces cerevisiae antibodies (ASCA) were tested and came back positive, both IgG antibody and IgA antibody. These findings confirmed the diagnosis of CD. Steroid treatment initially caused uncontrolled hyperglycemia, prompting a switch to budesonide.

The patient improved in regards to symptoms and was able to tolerate a diet and start to produce bowel movements. The patient was discharged with outpatient GI follow-up for immunomodulatory treatment.

In this case, CT imaging revealed thickening of the terminal ileum, a diagnostic challenge that requires comprehensive evaluation. The patient was a Caucasian male. CD and intestinal tuberculosis (ITB) are the primary considerations in such cases. However, the patient's late-stage ascites without lung involvement, lumped abdomen, fever, and night sweats, along with negative QuantiFERON test and chest X-ray findings ruled out ITB. The presence of terminal ileitis on CT imaging and a positive ASCA further supported the diagnosis of CD. Human leukocyte antigen (HLA)-A and HLA-B were not tested.

This atypical presentation of CD deviates from the typical symptom progression, highlighting the importance of recognizing atypical symptoms and early intervention. Recognizing such cases is crucial for optimizing patient outcomes, as the underlying cause can range from benign to life-threatening conditions. Further research is needed to refine diagnostic techniques and treatment approaches for these challenging clinical scenarios.

## Discussion

The unexpected diagnosis of CD in a 70-year-old patient presenting with initial symptoms of SBO prompts a multifaceted discussion, encompassing the diagnostic challenges, the atypical nature of the presentation in this age group, and the implications for management and care in elderly individuals. Our case provides an example of this, where further investigation of an SBO can lead to diagnosing CD, which is atypical.

CD is a complex inflammatory disorder believed to result from immune dysregulation and dysbiosis, leading to chronic inflammation with an unclear cause [8]. Risk factors include familial history, genetic predisposition, such as the HLA-B27 association, and tobacco use [9]. CD can affect individuals at various ages, with peak incidence occurring in two age groups, 15-35 and 55-70 years [10]. Our patient had no family history and was at the tail end of the age demographic.

Symptoms of CD can be insidious and nonspecific, including weight loss, low-grade fevers, and fatigue [11]. Chronic diarrhea, malabsorption, and extraintestinal complications like enterocutaneous or perianal fistulas are common [11]. Symptoms may precede diagnosis by several years. Bowel obstructions in CD present with abdominal pain, lack of flatus and bowel movements, hyperactive bowel sounds, and nausea and vomiting [11]. Patients with penetrating CD can develop fistulas or abscesses. Elderly CD patients can be without the hallmark symptoms of CD, making diagnosis challenging in the elderly population group [12]. Our patient only had signs of bowel obstruction.

A previous study also reported an elderly patient who presented with a months-long history of atypical symptoms involving three months of poor appetite, intermittent fecal urgency, and loose stools with mucus discharge [12]. The patient had three previous routine screening colonoscopies many years ago that showed non-specific colitis, with the most recent colonoscopy showing granuloma according to the patient, but the patient failed to follow up [12]. A colonoscopy on admission showed ulceration, acute cryptitis, lamina propria, and increased chronic inflammation with crypt architectural distortion, given these findings and the patient’s history, CD was diagnosed and treatment was initiated for the patient [12]. Our patient’s previous colonoscopies were all within normal limits.

Elderly individuals with CD frequently exhibit atypical clinical features compared to younger cohorts. The altered immune response and the presence of multiple comorbidities in the elderly can obscure the classic signs of Crohn's, leading to delayed or missed diagnoses [12]. In this context, recognizing the diverse and subtle presentations of Crohn's in older patients becomes imperative [13].

The histopathological confirmation of CD often necessitates endoscopic evaluation or surgical intervention. In the context of obstructive symptoms, endoscopic assessment might have been deferred due to the perceived risk, opting instead for a more conservative approach or exploratory surgery. This highlights the challenges of obtaining definitive histological confirmation in cases presenting with acute obstruction.

The management of CD in the elderly poses unique considerations. Due to the lack of studies conducted on treatment for elderly CD patients, the current treatment principles follow that of younger CD patients [14]. In the elderly population, it is important to take into consideration additional factors such as diabetes mellitus (DM), hypertension, cardiovascular diseases, smoking history, renal dysfunction, and functional and cognitive impairments when choosing treatment options [15]. Treatment strategies must balance disease control with the heightened susceptibility to medication-related adverse effects in this age group. Moreover, polypharmacy commonly observed in the elderly necessitates a tailored approach to therapy [15].

Therapeutic decisions likely involve a multidisciplinary team comprising gastroenterologists, surgeons, and geriatric specialists. The utilization of corticosteroids, immunomodulators, or biologic agents might be approached cautiously in consideration of the patient's age-related vulnerabilities [14].

## Conclusions

This case report elucidates the diagnostic intricacies encountered in a 70-year-old patient initially presenting with signs suggestive of SBO, ultimately leading to the unexpected diagnosis of CD. The patient failed to follow up outpatient with the gastroenterologist and instead had a return of symptoms and hospitalization. By highlighting this atypical presentation in an elderly individual, the report aims to underscore the need for heightened vigilance, comprehensive assessments, and a reconsideration of diagnostic paradigms in geriatric patients with GI symptoms suggestive of obstruction. Prompt diagnosis and a multidisciplinary approach involving gastroenterologists and surgeons are crucial in optimizing patient outcomes. Conservative management with corticosteroids may be successful in selected cases such as this, but surgery should be considered when there is no clinical improvement or if signs of bowel ischemia or perforation develop. Long-term management and surveillance are essential in elderly patients with CD to prevent complications and improve their quality of life.
